# NF-kappaB Is Involved in the Regulation of EMT Genes in Breast Cancer Cells

**DOI:** 10.1371/journal.pone.0169622

**Published:** 2017-01-20

**Authors:** Bruno R. B. Pires, Andre L. Mencalha, Gerson M. Ferreira, Waldemir F. de Souza, José A. Morgado-Díaz, Amanda M. Maia, Stephany Corrêa, Eliana S. F. W. Abdelhay

**Affiliations:** 1 Laboratório de Célula-Tronco, Instituto Nacional de Câncer José Alencar Gomes da Silva, Rio de Janeiro, RJ, Brazil; 2 Instituto Nacional de Ciência e Tecnologia para o Controle do Câncer, Rio de Janeiro, RJ, Brazil; 3 Departamento de Biofísica e Biometria, Universidade do Estado do Rio de Janeiro, Rio de Janeiro, RJ, Brazil; 4 Grupo de Biologia Estrutural, Instituto Nacional de Câncer José Alencar Gomes da Silva, Rio de Janeiro, RJ, Brazil; University of Alabama at Birmingham, UNITED STATES

## Abstract

The metastatic process in breast cancer is related to the expression of the epithelial-to-mesenchymal transition transcription factors (EMT-TFs) SNAIL, SLUG, SIP1 and TWIST1. EMT-TFs and nuclear factor-κB (NF-κB) activation have been associated with aggressiveness and metastatic potential in carcinomas. Here, we sought to examine the role of NF-κB in the aggressive properties and regulation of EMT-TFs in human breast cancer cells. Blocking NF-κB/p65 activity by reducing its transcript and protein levels (through siRNA-strategy and dehydroxymethylepoxyquinomicin [DHMEQ] treatment) in the aggressive MDA-MB-231 and HCC-1954 cell lines resulted in decreased invasiveness and migration, a downregulation of SLUG, SIP1, TWIST1, MMP11 and N-cadherin transcripts and an upregulation of E-cadherin transcripts. No significant changes were observed in the less aggressive cell line MCF-7. Bioinformatics tools identified several NF-κB binding sites along the promoters of SNAIL, SLUG, SIP1 and TWIST1 genes. Through chromatin immunoprecipitation and luciferase reporter assays, the NF-κB/p65 binding on TWIST1, SLUG and SIP1 promoter regions was confirmed. Thus, we suggest that NF-κB directly regulates the transcription of EMT-TF genes in breast cancer. Our findings may contribute to a greater understanding of the metastatic process of this neoplasia and highlight NF-κB as a potential target for breast cancer treatment.

## Introduction

Breast cancer is the leading cause of cancer death among women worldwide. According to GLOBOCAN, this neoplasia is responsible for 522,000 deaths in women each year [1]. Late diagnosis increases the risk of cancer cells spreading from the primary tumor to neighboring tissues and distant organs in a process known as metastasis. In breast cancer, almost all deaths are attributed to metastasis, which is responsible for 90% of deaths from solid tumors [2]. Metastasis involves sequential and interrelated steps: cancer cells develop an invasive growth, detach from the primary tumor, degrade the basement membrane, migrate into the circulatory system (intravasation), evade immune attack, and escape from capillaries (extravasation) to invade and proliferate in distant organs [3].

The epithelial-to-mesenchymal transition (EMT) is considered one of the major mechanisms involved in solid tumor metastasis. During the EMT, tumor cells lose their epithelial features and acquire a mesenchymal phenotype leading to invasive and migratory behavior. The earliest events of the EMT include the downregulation of cell-cell adhesion molecules, such as the adherens junction protein E-cadherin, and upregulation of weak adhesion related molecules, such as N-cadherin and vimentin. In addition, matrix metalloproteinases (MMPs), which are capable of degrading the extracellular matrix (ECM), are upregulated. This phenotype switch is a reversible process regulated mostly by the transcription factors *SNAIL1*, *SLUG (SNAIL2)*, *TWIST1* and *SIP1 (ZEB2)*, which downregulate epithelial markers and upregulate mesenchymal markers and MMPs [4–9]. Moreover, overexpression of these transcriptional factors in breast cancer has been correlated with higher histologic grade, lymph node metastasis and poor survival [10–12].

The intense cell migration and invasion during metastasis have characteristics common with those of cell movements during embryogenesis. During *Drosophila* sp. development, dorsal acts as a transcriptional activator of *snail* and *twist*, which are crucial to gastrulation [13,14]. The mammalian ortholog of *dorsal* is the nuclear factor-κB (NF-κB) family. However, the regulatory mechanisms of this modulation have not yet been demonstrated in human cancers.

The NF-κB family has been described as a critical regulator of a large number of biological processes, including cell proliferation, differentiation, immune responses and inflammation. This family consists of five subunits: p50 (NF-κB1), p52 (NF-κB2), p65 (RelA), c-Rel (Rel) and RelB, which associate to form functional homo- and heterodimers. The NF-κB complex is usually inactive and located in the cytoplasm while bound to IκB inhibitor proteins. For the NF-κB complex to be released from its inhibitor, the IκB protein must be phosphorylated by the IκB kinase (IKK) complex, which leads to IκB ubiquitination and subsequent degradation by the 26S proteasome. NF-κB is then translocated into the nucleus and activates gene transcription by binding to sequence-specific target DNA [15] known as κB sites (5′-GGGRNYYYCC-3′, where R: purine, Y: pyrimidine and N: any nucleotide) [16]. This complex has more than 150 regulatory target genes, and the most abundant heterodimer found is the p65/p50 complex [17,18]. Recently, a positive correlation between EMT transcription factors and NF-κB activation has been described in several human cancers [19]: breast cancer [20], prostate cancer [21], renal carcinoma [22], and head and neck squamous cell carcinomas [23]. However, there is no evidence to date on how NF-κB regulates EMT-inducing factors.

Pharmacological and genetic approaches are commonly applied to study specific cell signaling. The NF-κB inhibitor DHMEQ (dehydroxymethylepoxyquinomicin), which is a derivative of the antibiotic epoxyquinomicin C [24], directly binds to NF-κB/p65 and specifically represses its nuclear translocation and its DNA-binding activity [25,26]. DHMEQ has a unique specificity in blocking NF-κB activity [15], and preclinical studies on cancer cell lines or in xenograft settings have shown effective growth inhibition in several tumors, such as breast cancer [27], prostate cancer [28], bladder cancer [29], thyroid cancer [30], pancreatic cancer [31], head and neck carcinoma [32], multiple myeloma [33] and adult T-cell leukemia [34]. Due to its chemical properties and biological activity, the use of DHMEQ has been promising for *in vitro* studies and clinical procedures. In this study, the specific properties of DHMEQ as an NF-κB/p65 inhibitor were utilized to specifically affect this transcriptional factor. Moreover, interference technology, such as siRNA, can also produce direct effects on one target and allow the evaluation of its downstream-regulated genes and phenotype.

Here, we sought to examine the impact of NF-κB inhibition on the tumoral properties of human breast cancer cells and to determine how NF-κB regulates the expression of EMT transcription factors. The inhibition of NF-κB (using DHMEQ and siRNA strategies) decreased EMT-TF levels in the aggressive cells. Moreover, a significant binding of NF-κB to the *SLUG*, *TWIST1* and *SIP1* promoter regions was observed, and the activation of these genes by NF-κB through promoter activity was confirmed using a luciferase reporter strategy. Altogether, our results provide evidence regarding NF-κB’s transcriptional involvement in EMT-TF regulation.

## Materials and Methods

### Cell lines and culture

The human breast cancer cell lines MCF-7 (ATCC HTB-22), HCC-1954 (ATCC CRL-2338) and MDA-MB-231 (ATCC HTB-26) were cultured in RPMI-1640 (Sigma-Aldrich) supplemented with 10% fetal bovine serum (FBS), 2 mM glutamine, 100 units/ml penicillin and 100 μg/ml streptomycin. The cells were cultured in a humidified 5% CO_2_ atmosphere at 37°C.

### Chemicals

DHMEQ, the NF-κB/p65 inhibitor, was synthesized as previously described [16]. All the cell lines studied were subjected to treatment with DHMEQ and evaluated in various conditions described in the manuscript.

### Cell viability assay

Different concentrations (3, 10 and 30 μg/ml) of DHMEQ were used to investigate the dose-response effect. We determined the cell viability with the colorimetric WST1 (water-soluble tetrazolium salt) kit according to the manufacturer’s instructions (Roche).

### Cell migration assay

To address the contribution of NF-κB/p65 to cancer cell migration, a wound healing assay was performed. Briefly, breast cancer cells were seeded into a 6-well plate and cultured as described above. Cell monolayers at 90% confluence were wounded by scratching them with a 200 μl plastic tip, washed with phosphate buffered saline (PBS), and incubated in fresh culture medium supplemented with 1% FBS to prevent proliferation [35] in the presence or absence of DHMEQ (10 μg/ml). Cell cultures were photographed (x100 magnification) 24 h after wounding using an Axio Observe.Z1 microscope equipped with an AxioCam HRc and the AxioVision Release 4.8 digital image processing software (Carl Zeiss Inc.). The relative width of the wounds was obtained as the average distance between edges, and the original wound width (0 h) was defined as 100% [35]. The experiments were performed in triplicate.

### Cell invasion assay

A Matrigel transwell invasion assay was performed to examine the effect of NF-κB/p65 inhibition on the cell invasion process. Approximately 10^4^ cells were seeded in medium containing 1% FBS in the presence or absence of DHMEQ (10 μg/ml) onto the upper region of the transwell chamber (8-μm pore size; Corning) coated with a Matrigel basement membrane-like matrix (1 mg/ml; BD). The lower chamber of the transwell was filled with medium containing 10% FBS. After a 24-h incubation at 37°C and 5% CO_2_, the cells were fixed with absolute ethanol and stained with crystal violet, and the non-invaded cells present in the inserts were removed with a cotton swab. Images of the invaded cells were acquired using the same equipment described above. Five random fields were photographed (x200 magnification) and counted. The values of non-treated cells were defined as 100%, and the relative proportion was calculated for DHMEQ-treated cells. The experiments were performed in triplicate.

### Real-time reverse transcription polymerase chain reaction (RT-qPCR) analysis

The mRNA levels of cells were investigated by RT-qPCR. Briefly, total RNA was purified using the TRIzol reagent (Invitrogen) according to the manufacturer’s instructions. Then, 2 μg of RNA was processed using the DNase Amplification Grade I Kit (Invitrogen) to remove DNA contamination and was reverse transcribed into cDNA using the Superscript-III First Strand Synthesis kit (Invitrogen) following the manufacturer’s protocol. RT-qPCR was performed with the SYBR Green Master Mix (Invitrogen) in a Rotor-Gene Q (Qiagen), and the conditions were as follows: 95°C for 10 min, followed by 40 cycles of 30 s at 95°C, 30 s at 60°C and 30 s at 72°C. Each sample was examined in triplicate. The primers used are described in S1 Table. *ACTB* and *GAPDH* were used as the reference genes for the mRNA levels. Fold-expression was calculated according to the ΔΔC_t_ method [36].

### Prediction of NF-κB binding regions

To screen for putative NF-κB binding sites along 1,000 bp upstream of the transcription start site of *SNAIL1*, *SLUG*, *SIP1* and *TWIST1* genes, we used on-line prediction tools. The on-line software used included the following: Transfac (http://www.gene-regulation.com), Tfsitescan (http://www.ifti.org/cgi-bin/ifti/Tfsitescan.pl), TESS (http://www.cbil.upenn.edu/cgi-bin/tess/tess), TFBind (http://tfbind.hgc.jp/) and TFSearch (http://www.cbrc.jp/research/db/TFSEARCH.html). The DNA sequences of the promoter regions were acquired from the NCBI database (www.ncbi.nlm.nih.gov). The alignment among species was performed using the Ensembl orthology tool (www.ensembl.org).

### Chromatin immunoprecipitation (ChIP)

To confirm the predicted NF-κB binding sites, ChIP assays were conducted as described previously [37]. Briefly, the lysed and digested material was incubated with 5 μg of NF-κB/p65 antibody (C-20, sc-372, Santa Cruz Biotechnology) or with a negative immunoprecipitation control normal rabbit IgG (#2729, Cell Signaling). We next purified the DNA using microcolumns and subjected it to qPCR, employing specific primers for each putative NF-κB binding site listed in S2 Table. Reactions were performed under the same conditions as described above. The fold-change of the NF-κB-precipitated sample was calculated in relation to the IgG-precipitated control. Both samples were normalized by the input C_t_ [38]. Each sample was examined in triplicate.

### Luciferase reporter assay

The plasmids used for this experiment were as follows: pGL3-Promoter Vector, pGL3-plasmid containing *SLUG*, *SIP1* or *TWIST1* promoter regions (cloned as described below) and an internal control pRL-TK renilla luciferase expression plasmid (Promega). Each DNA promoter region was amplified by PCR from human genomic DNA with the primers listed in S2 Table. For promoter constructs without predicted sites, we used the following primers: *SLUG* promoter -398 bp forward 5'-GGCTCTCATTAACACCAGAGG-3' and +5 bp reverse 5'-CCTTTACGAACTGAGCCCG-3'; *TWIST1* promoter -38 bp forward 5'-TCCTCCTCACGTCAGGC-3' and +29 bp reverse 5’-GTCTGGGAGTTGGGCGAGA-3’; and SIP1 promoter -636 bp forward 5'-TCCCTGCTAAGTTTCTCTATGGC-3' and +22 bp reverse 5'-CCTTGAAGTCTCCGCAAACG-3'. The PCR products were inserted into the PCR 2.1 TOPO plasmid (Invitrogen). Next, they were digested with XhoI and SacI (Promega) and inserted into the corresponding sites of pGL3-Promoter Vector using T4 DNA ligase (Invitrogen). We co-transfected 0.2 μg of pGL3-Promoter Vector or pGL3-DNA promoter with 0.2 μg of pRL-TK Renilla plasmid in MDA-MB-231 cells with Lipofectamine LTX with Plus Reagent (Invitrogen). The extracts were prepared from cells 48 h after transfection of the constructs using a Dual-Luciferase Reporter Assay System (Promega) to measure luciferase enzyme activity in a Veritas Microplate Luminometer (Turner BioSystems) according to the manufacturer’s instructions. Luciferase activity was expressed as relative light units, the firefly luciferase was normalized to the renilla vector, and the values were reported relative to the pGL3 (Mock) signal. All of the experiments were performed in triplicate.

### NF-κB/p65 knockdown

To knockdown NF-κB/p65 *in vitro*, specific short interference RNA (siRNA) was used (sc-29410, Santa Cruz Biotechnology). A siRNA that does not match any known human coding cDNA was used as a negative control for silencing (Scramble, sc-37007, Santa Cruz Biotechnology). Transfections were performed using Lipofectamine LTX (Invitrogen) as per the manufacturer’s instructions, 4 × 10^5^ cells were plated in a 6-well plate containing RPMI-1640 media without antibiotics, and transfections were conducted with 50 nM of NF-κB/p65 siRNA (or Scramble) for up to 72 h.

### Immunoblotting

Whole-cell protein extracts were obtained from cell lines in lysis buffer containing 50 mM Tris pH 7.5, 5 mM EDTA, 10 mM EGTA, 50 mM NaF, 20 mM b-glycerolphosphate, 250 mM NaCl, 0.1% Triton X-100, 20 mM Na_3_VO_4_ and protease inhibitor mix (Promega). The protein concentrations were determined using the Bradford assay, and 30 μg of the cell lysate proteins was separated by 12% sodium dodecyl sulfate-polyacrylamide gel electrophoresis (SDS-PAGE). The proteins were transferred to nitrocellulose membranes (Bio-Rad), blocked with 5% milk and incubated with the anti-NF-κB/p65 (ab16502-100, Abcam) and anti-Slug (C19G7, Cell Signaling) antibodies at 1:1000 dilutions. Antibody binding was detected using ECL reagents (Thermo Scientific). Images were acquired using Image Studio Digits software v 3.1 with a LI-COR instrument (Uniscience). Rouge Ponceau staining was used to assess equal loading.

### Statistical analysis

All of the data were expressed as the mean ± standard deviation (SD) of at least three independent experiments and analyzed by a two-tailed Student's t-test using GraphPad Prism v.5 (GraphPad Inc., USA). *p*-values <0.05 were considered statistically significant.

## Results

### NF-κB/p65 inhibition decreases the malignant potential of breast cancer cells

DHMEQ was used to inhibit NF-κB/p65 activity through its binding and repression of nuclear translocation and its DNA-binding activity. Initially, we evaluated the dose-response of DHMEQ in the cell lines used in this study through a cell viability assay (WST1 kit, Roche) and an NF-κB/p65-luciferase reporter assay (we performed a luciferase reporter assay, as described in the Materials and Methods section, using a construct containing NF-κB response elements fused to the luciferase gene [39]) (S1 and S2 Figs, respectively). DHMEQ at 10 μg/ml inhibited NF-κB activity without significantly affecting the cell viability. Therefore, this concentration was used in all of the experiments.

**Fig 1:**
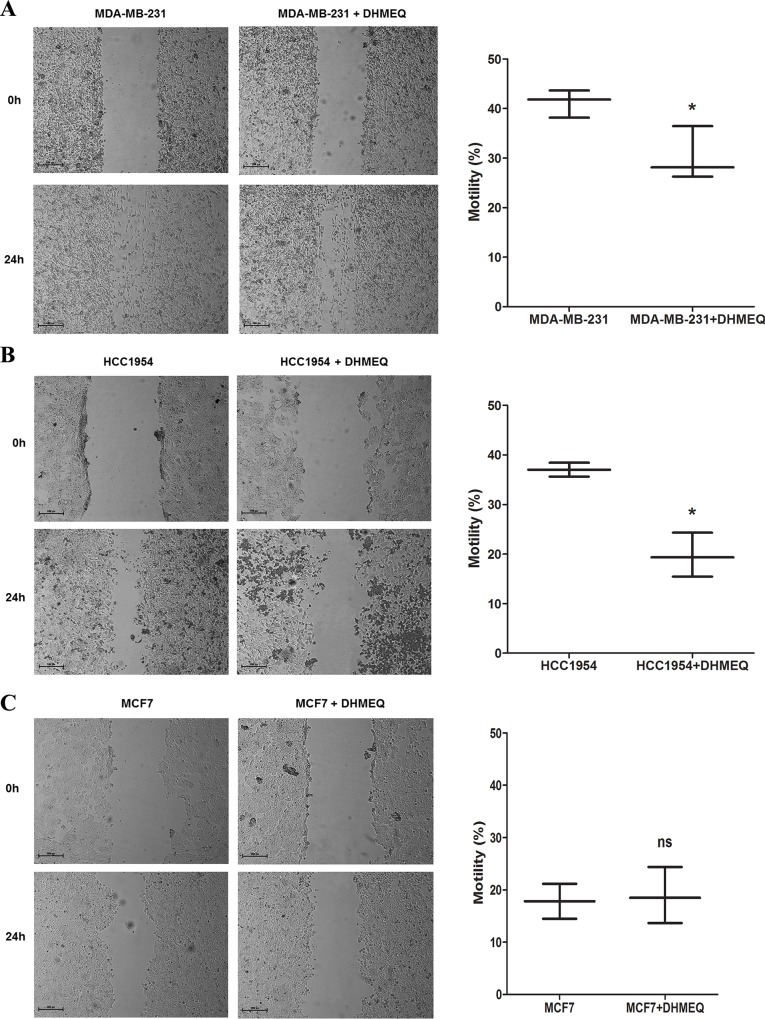
Migration assay. A representative wound healing assay evaluating cell migration at 24 h after DHMEQ treatment of MDA-MB-231 (A) HCC-1954 (B) and MCF-7 (C) cells is shown. The box plots represent migratory ability as indicated by the percent of wound closure. Magnification x100. The data were expressed as the mean ± SD. * = *p*<0.05, ns = not statistically significant.

**Fig 2:**
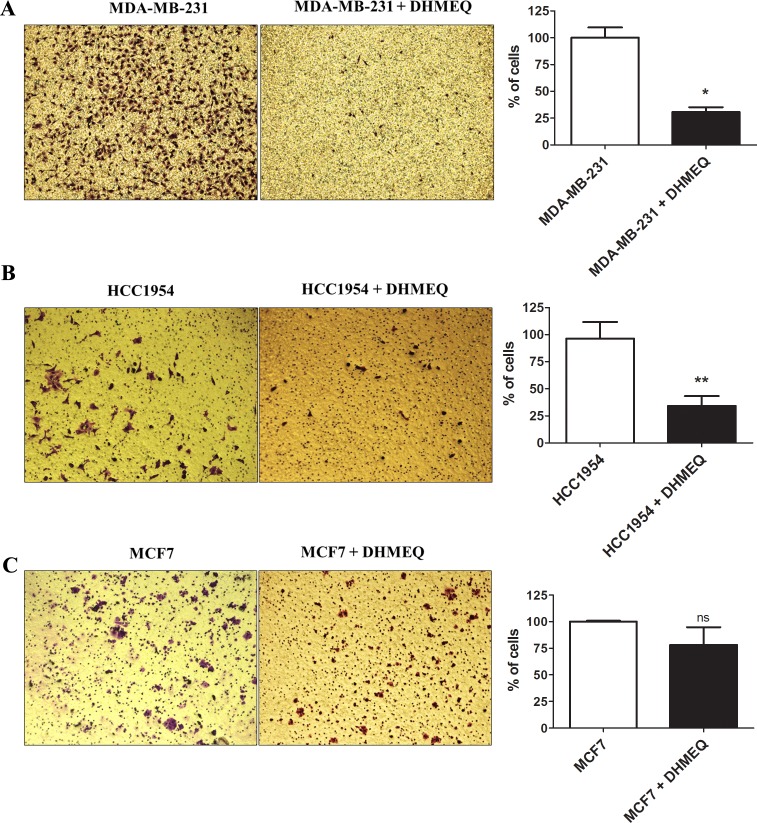
Invasiveness assay. A representative Matrigel transwell assay evaluating invasive potential at 24 h after DHMEQ treatment of MDA-MB-231 (A) HCC-1954 (B) and MCF-7 (C) cells is shown. The bar graph represents the relative invasive potential of MDA-MB-231, HCC-1954 and MCF-7 cells. The cells were stained with crystal violet. Magnification x200. The data were expressed as the mean ± SD. * = *p*<0.05, ** = *p*<0.01, ns = not statistically significant.

To evaluate the role of NF-κB/p65 in the regulation of the malignant potential of breast cancer cells, the cells were treated with DHMEQ (10 μg/ml), and wound healing migration and Matrigel transwell invasion assays were subsequently conducted. As seen in Fig 1, treatment with DHMEQ decreased the motility of MDA-MB-231 (Fig 1A) and HCC-1954 (Fig 1B) cells (1.36- and 1.88-fold, respectively). Furthermore, an important reduction of their invasive potential (3.26- and 2.86-fold, respectively) was observed (Fig 2A and 2B). Nevertheless, the migratory and invasive potential of MCF-7 cells was not significantly affected after treatment with DHMEQ (Figs 1C and 2C). Together, these data suggest that NF-kB/p65 can influence the aggressive features in breast cancer cells.

### NF-κB/p65 inhibition reduces SLUG, TWIST1 and SIP1 expression and reverts the EMT expression pattern

**Fig 3:**
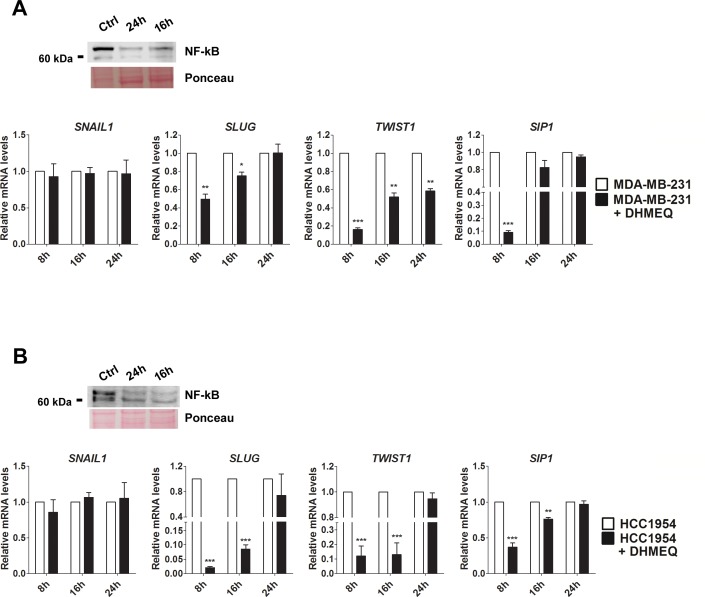
Relative expression of the EMT-inducing factors after NF-κB/p65 signaling inhibition. The mRNA levels of *SNAIL1*, *SLUG*, *TWIST1*, and *SIP1* were assessed in MDA-MB-231 (A) and HCC-1954 (B) cells at 8, 16 and 24 h of DHMEQ treatment. NF-κB/p65 inhibition was evaluated at protein levels by western blot assay at 16 and 24 h of DHMEQ treatment. Ponceau staining was used as a loading control. Ctrl: control. The data were expressed as the mean ± SD. * = *p*<0.05, ** = *p*<0.01, *** = *p*<0.001.

**Fig 4:**
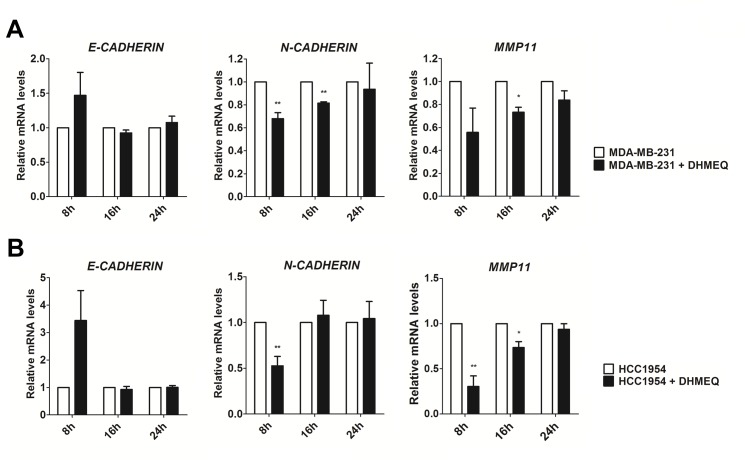
Relative expression of EMT-phenotype markers after NF-κB/p65 signaling inhibition. The mRNA levels of *E-CADHERIN*, *N-CADHERIN* and *MMP11* were assessed in MDA-MB-231 (A) and HCC-1954 (B) cells at 8, 16 and 24 h of DHMEQ treatment. The data were expressed as the mean ± SD. * = *p*<0.05, ** = *p*<0.01, *** = *p*<0.001.

**Fig 5:**
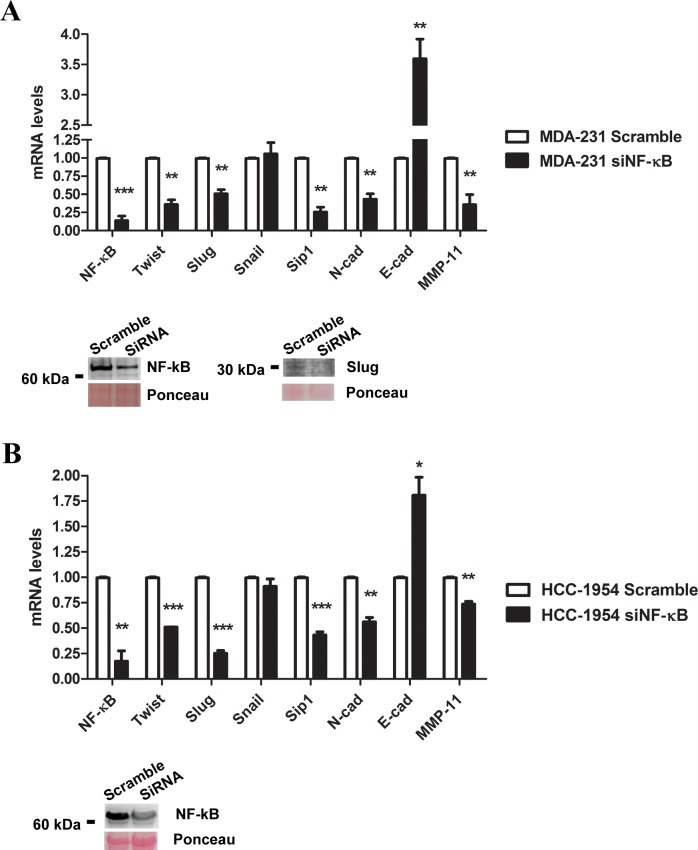
Relative expression of EMT-related genes after NF-κB/p65 genetic silencing using a siRNA approach. The mRNA levels of the EMT-inducing factors *SNAIL1*, *SLUG*, *TWIST1*, and *SIP1* and EMT-phenotype markers *E-CADHERIN*, *N-CADHERIN* and *MMP11* together with NF-κB/p65 inhibition at the protein level were assessed in MDA-MB-231 (A) and HCC-1954 (B) cells (scramble and siNF-κB/p65). Moreover, Slug expression was evaluated at the protein level for MDA-MB-231 by western blot assay in scramble and siNF-κB cells. Ponceau staining was used as a loading control. The data were expressed as the mean ± SD. * = *p*<0.05, ** = *p*<0.01, *** = *p*<0.001.

As our experiments indicated that NF-κB/p65 is important for the cell migratory and invasive properties of breast cancer cells (Figs 1 and 2), we questioned whether NF-κB/p65 inhibition would alter EMT-related gene expression. For this purpose, we conducted pharmacological inhibition of NF-κB through DHMEQ treatment in MDA-MB-231, HCC-1954 and MCF-7 cells. We found that *SLUG*, *TWIST1* and *SIP1* transcript expression was decreased at 8 h of DHMEQ treatment in MDA-MB-231 cells (Fig 3A) and HCC-1954 (Fig 3B). Our findings also showed an upregulation of the epithelial marker *E-CAD* and downregulation of *N-CAD* and *MMP11* for both cell lines (Fig 4A and 4B). To further strengthen our data and evaluate a direct role of NF-κB, we performed NF-κB knockdown in the same cell models. As shown in Fig 5, the gene expression profile obtained for *SLUG*, *TWIST* and *SIP1* after pharmacological inhibition of NF-κB was also observed after its silencing through siRNA, further suggesting that NF-κB supports the EMT expression pattern. *SNAIL1* expression was not significantly altered for any condition in any cell line. Moreover, no significant changes were observed in the less aggressive cell line MCF-7 (S3A, S3B and S3C Fig).

NF-κB protein expression was evaluated in both strategies applied to silence its signaling. As shown in Fig 3 and Fig 5, a reduction in NF-κB/p65 expression was observed for both cell lines, suggesting a downregulation in the signaling pathway. Additionally, the downstream target SLUG was also evaluated, and its expression was reduced in siNF-κB MDA-MD-231 cells (Fig 5A).

### *In silico* determination and ChIP confirmation of NF-κB binding sites in the SLUG, TWIST1 and SIP1 promoter regions

**Fig 6:**
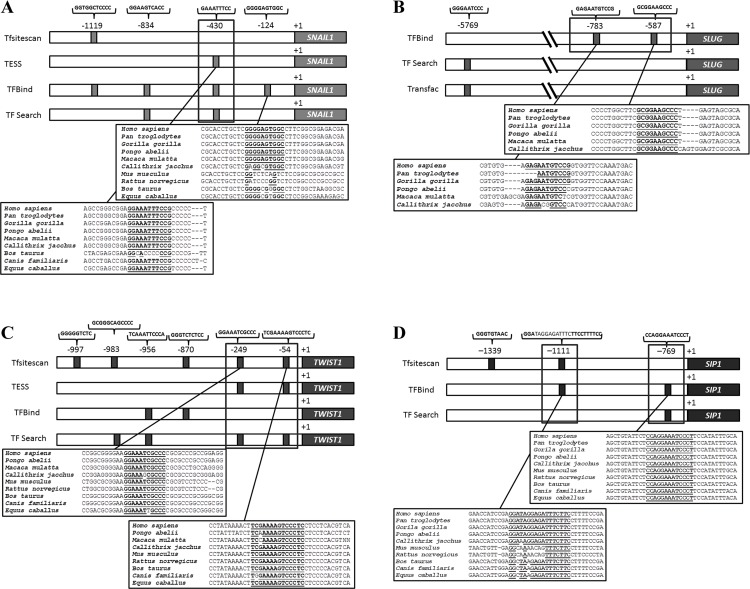
**Representative scheme of putative NF-κB binding sites located in the SNAIL1 (A), SLUG (B), TWIST1 (C) and SIP1 (D) promoter regions predicted by Tfsitescan, TESS, TFBind, TFSearch and Transfac bioinformatics tools.** An alignment of the DNA region showed evolutionarily conservation among metazoan species. Identical nucleotides are in bold. Gray lines indicate regions investigated by chromatin immunoprecipitation. +1: transcription start site.

To predict κB sites in the promoter regions of the previously investigated EMT-inducing factors, we used several bioinformatics tools. We identified four NF-κB binding consensus sequences in the *SNAIL* promoter (-124, -430, -834 and -1119 bp) (Fig 6A), two in the *SLUG* promoter (-587 and -783 bp) (Fig 6B), six in the *TWIST1* promoter (-54, -249, -870, -956, -983 and -997 bp) (Fig 6C) and three in the *SIP1* promoter (-769, -1111 and -1339 bp) (Fig 6D). Alignment analyses revealed that the proximal regions were highly conserved among metazoan species.

**Fig 7:**
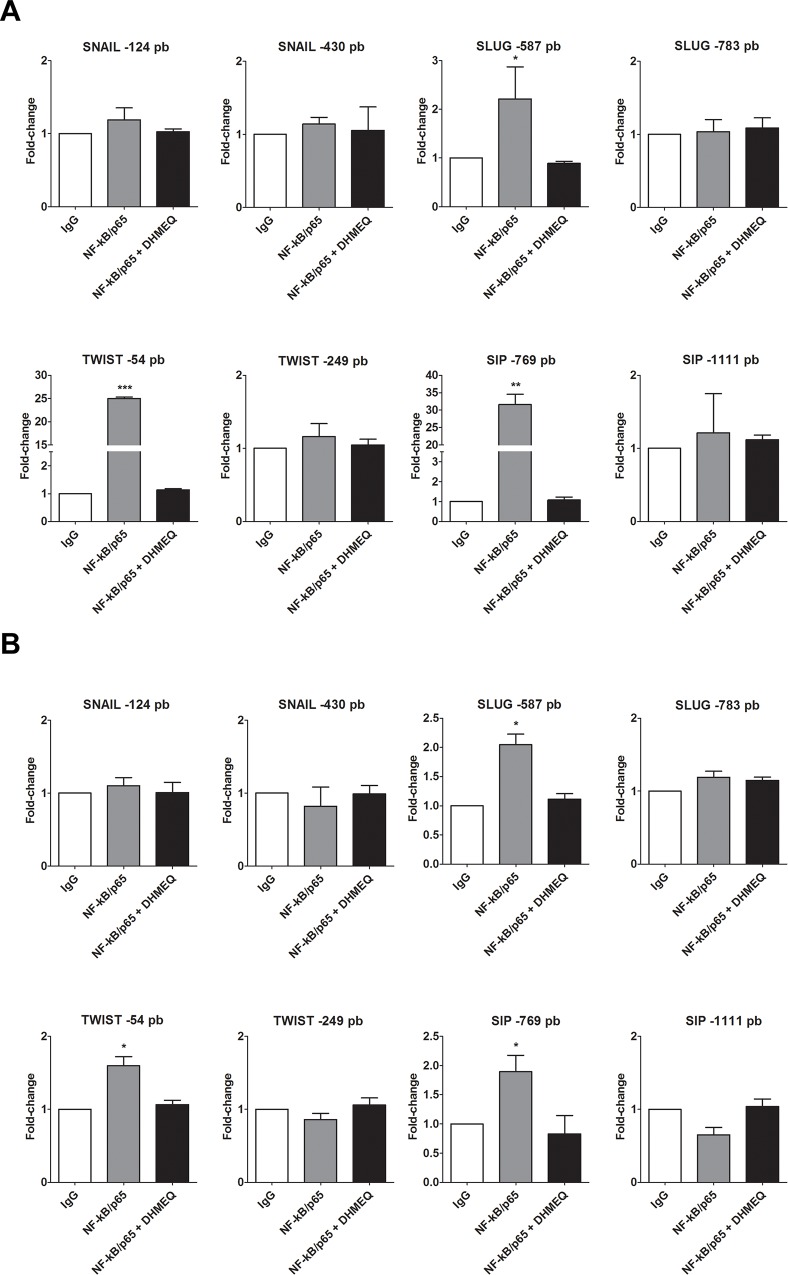
**ChIP-qPCR of predicted NF-κB/p65 binding sites in the SNAIL1, SLUG, TWIST1 and SIP1 promoter regions using MDA-MB-231 (A) and HCC-1954 (B) cells.** The histograms set a fold-change of each site by comparing the IgG negative control to NF-κB/p65 antibodies with the natural and treated (DHMEQ) condition. The data were expressed as the mean ± SD. * = *p*<0.05, ** = *p*<0.01, *** = *p*<0.001.

To address whether NF-κB binds directly to the predicted sites in the *SNAIL1*, *SLUG*, *TWIST1* and *SIP1* promoters, we performed a ChIP assay followed by qPCR (ChIP-qPCR) using the MDA-MB-231 (Fig 7A), HCC-1954 (Fig 7B) and MCF-7 (S3C Fig) cell lines. Consistent with the mRNA levels of EMT-inducing factors, a significant binding of NF-κB to *SLUG* -587 bp, *TWIST1*–54 bp and *SIP1*–769 bp sites was observed in both the MDA-MB-231 and HCC-1954 cell lines. No predicted site in the *SNAIL1* promoter region was shown to be an NF-κB binding site, and the same result was found for the *SLUG* -783, *TWIST1*–249 and *SIP1*–1111 bp sites in all of the studied cell lines. Then, the NF-κB inhibitor was used to confirm the specificity of NF-κB binding to the selected sites. DHMEQ treatment decreased the enrichment of all of the sites tested in the ChIP samples, resulting in signals similar to the basal levels found in the IgG negative control (Fig 7). ChIP assays performed in MCF-7 cells did not show significant binding of NF-κB to any of the predicted sites (S3C Fig). These observations confirmed the *TWIST1* promoter -54 bp, *SLUG* promoter -587 bp and *SIP1* promoter -769 bp sites as direct transcriptional targets of NF-κB in aggressive breast cancer cells.

### Activation of the SLUG, TWIST1 and SIP1 promoters by NF-κB

**Fig 8:**
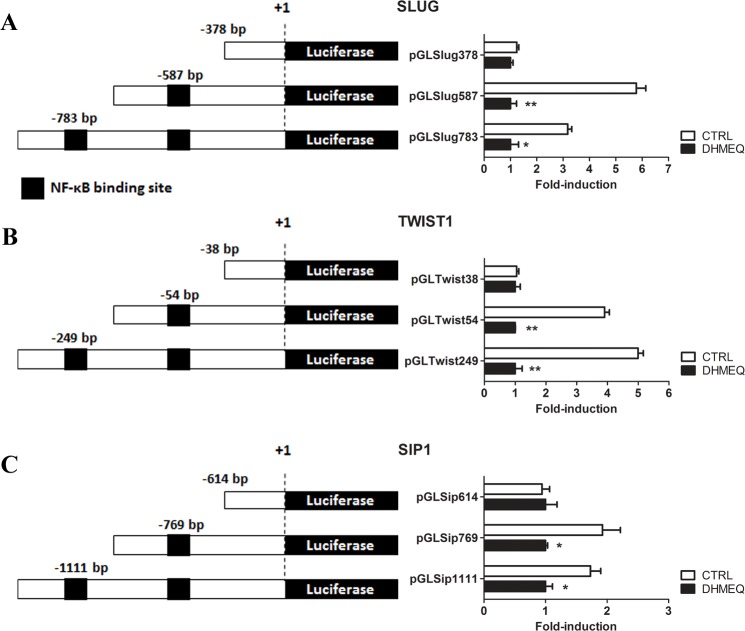
**Relative luciferase activity in MDA-MB-231 cells transfected with pGL3-plasmid containing the SLUG (A), TWIST1 (B) and SIP1 (C) promoter regions.** The firefly luciferase was normalized to the renilla vector, and the values are relative to the pGL3 (Mock) signal. The black boxes in the schematic representation plasmid constructs represent NF-κB binding sites. The bar graphs represent the relative luciferase activities of each construct in MDA-MB-231 cells, the white bars indicate natural NF-κB expression, and the black bars show NF-κB inhibition through DHMEQ treatment (10 μg/ml for 16 h). Each bar represents the mean ± SD.

We examined *SLUG*, *TWIST1* and *SIP1* promoter activity using a luciferase reporter plasmid containing a proximal promoter fragment upstream of the luciferase gene. These constructs were transfected transiently into MDA-MB-231 cells with or without DHMEQ. As shown in Fig 8, we observed an increase in the promoter activity of SLUG -587, TWIST1–54 and SIP -769 when addressing NF-κB binding sites, suggesting the participation of NF-κB as a regulator of these EMT-TFs’ expression. These results were confirmed by a decrease in luciferase activity in DHMEQ-treated cells. These results corroborate the sites defined by ChIP as NF-κB binding sites.

## Discussion

The EMT is described as critical for the early stages of metastasis by regulating the cellular morphology and the gain of invasive/migratory potential by cancer cells [40–42]. Another property of cells undergoing the EMT is the secretion of proteases, such as MMPs, which cleave the protein components of the ECM, favoring migration. Concomitantly, the upregulation of mesenchymal markers, such as N-cadherin, and downregulation of E-cadherin provide a spindle shape to tumor cells, which facilitates their interaction with the ECM during the invasion of adjacent tissues [40,41]. It is noteworthy that after reaching their secondary site, cells undergo the mesenchymal-to-epithelial transition (MET) and acquire an epithelial phenotype similar to that of the primary tumor [40]. This process is characterized by a high phenotypic plasticity, which can be triggered by signals from the microenvironment [40–42].

The role of EMT-TFs has been consistently demonstrated in both xenograft models and human tumors [41]. In breast cancer, the role of these factors has been described during progression and metastasis. In addition, increased levels of SNAIL, SLUG, TWIST1 and SIP1 expression are related to poor prognosis and a higher risk of recurrence [10–12, 41].

We characterized NF-κB/p65 as a transcriptional regulator of the main EMT-inducing factors related to metastatic progression in breast cancer: SLUG, SIP1 and TWIST1. These results were corroborated by inhibition of NF-κB/p65 with DHMEQ, which reduced the migration and invasiveness of the aggressive breast cancer cells MDA-MB-231 and HCC-1954. Inhibition of NF-κB/p65 also changed the pattern of expression of EMT markers in both cell lines, resulting in reduced levels of SLUG, SIP1, TWIST1, N-cadherin and MMP11 concomitant with augmented levels of the epithelial marker E-cadherin. Indeed, NF-κB inhibition usually results in a reduction of the aggressive features of breast cancer, as demonstrated by others [43–45]. Our findings also support the potential of NF-κB inhibition for breast cancer treatment, which was intensively studied by Umezawa’s group through both *in vitro* [46] and *in vivo* [27] experiments.

Consistent with previous studies using human cancer models [47–49], our results showed that NF-κB inhibition was unable to change the expression of Snail in human breast carcinoma cells (Fig 3), although a correlation between these transcription factors has been demonstrated during the embryonic development of *Drosophila* sp. [13], *Xenopus laevis* [50] and *Danio rerio* (zebrafish) [51].

NF-κB inhibition not only decreased the motility and invasiveness of MDA-MB-231 and HCC-1954 cells (Figs 1 and 2) but also reduced the expression of EMT markers in a significant manner (Figs 3 and 4). In contrast to the results obtained for the aggressive cell lines, MCF-7 cells (which are less aggressive than the other cell lines) treated with DHMEQ showed no change in the migratory and invasiveness cellular assays or in the expression of EMT-associated genes (Figs 1 and 2 and S3 Fig). This finding can be explained by the fact that MCF-7 is described as expressing low levels of endogenous NF-κB [27] and EMT-inducing factors [7,52–54], which was confirmed by our group using RT-qPCR (data not shown).

The possible direct regulation of EMT-TFs by NF-κB has been discussed, although it has not been demonstrated experimentally to date. Huber et al. [43] demonstrated a strong correlation between NF-κB and EMT using a murine model of mammary carcinoma transfected with the oncogene c-Ras. These findings allowed the same authors [55, 56] to hypothesize that NF-κB might regulate EMT factors.

Computational predictions are an important tool to identify putative binding sites. Because different algorithms developed by different bioinformatics tools have been used, a great volume of data has been generated, which may show different or identical results. To solve this conflict, we focused on the more frequent and consensus sites among these bioinformatics tools. The predicted NF-κB binding sites were evolutionarily conserved among metazoan species, indicating a biological relevance of these regions throughout evolution (Fig 6). Furthermore, the data obtained by ChIP-qPCR assays (Fig 7) and confirmed by the luciferase promoter activity assays (Fig 8) showed strong evidence that NF-κB/p65 transcriptionally regulates the TWIST1 promoter at -54 bp, the SLUG promoter at -587 bp and the SIP1 promoter at -769 bp, which could be inhibited by treatment with DHMEQ. Consistent with these observations, NF-κB inhibition (through pharmacological and knockdown approaches) also decreased the mRNA levels of these three TFs (Figs 3 and 5), confirming them as NF-κB/p65 transcriptional targets. Interestingly, our western blot results showed diminished protein levels of NF-κB in DHMEQ treatment (Fig 3). This finding may be explained by the fact that NF-κB autoregulates its expression in a positive feedback loop [57]. As DHMEQ treatment pharmacologically inhibits NF-κB translocation and, consequently, activation, the observed effect could be occurring in breast cancer cells.

In contrast to previous work performed by Barberà et al. [58], our results showed no NF-κB/p65 binding in the Snail1 promoter region. Our differing findings are a result of methodological differences, as Barberà et al. [58] noted a putative NF-κB/p65 responsive region between -194 bp and -78 bp upstream of the transcription start site of this gene without describing the specific NF-κB binding site. We conducted a prediction analysis in the same region indicated by Barberà et al. [58] and found a likely binding site for NF-κB in the Snail1 promoter at -124 bp, but it was not confirmed by ChIP-qPCR (Fig 7). Later, the same group was unable to repeat this result in cancer models and claimed that the SNAIL promoter activation by NF-κB seems to be tissue specific [59]. Additionally, the former work and a study published by Katoh and Katoh [60] were not able to identify NF-κB binding sites in the SIP1 promoter, in contrast to our findings (Fig 6D). Regarding SLUG and TWIST1, Storci et al. [61] and Pham et al. [62], respectively, suggested a regulatory role of NF-κB in cancer models, but they did not experimentally demonstrate this mechanism.

**Fig 9:**
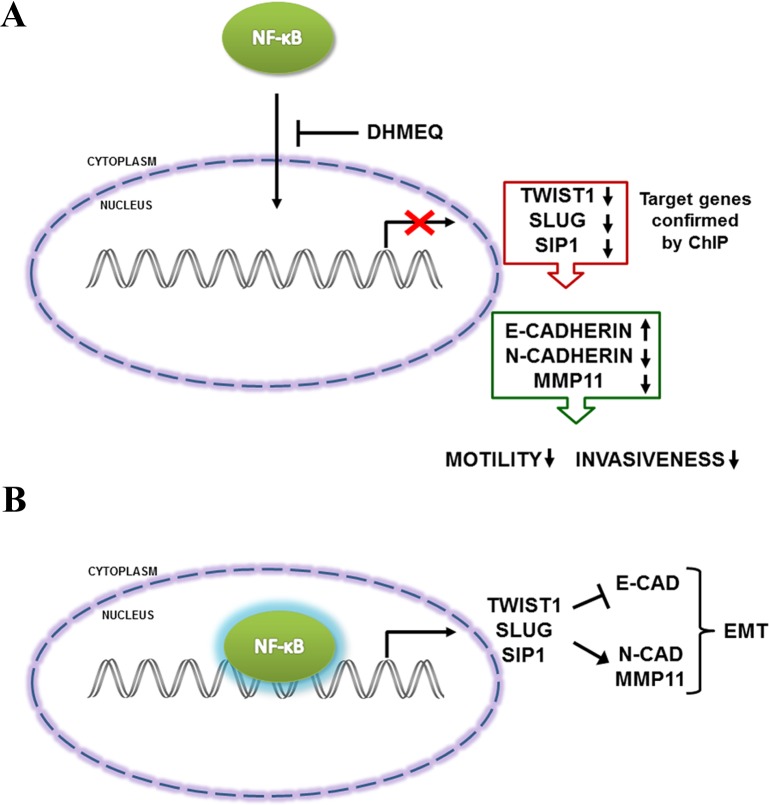
Schematic representation of our findings. A) Inhibition of NF-κB/p65 translocation reduces the expression of EMT-transcription factors that have been shown to regulate target genes of NF-κB. This inhibition increased E-cadherin expression, decreased N-cadherin and MMP11 expression and reduced cell motility and invasiveness potential. B) Then, NF-κB/p65 transcriptionally regulates the promoter regions of TWIST1, SLUG and SIP1, which in turn represses the epithelial marker E-cadherin and activates the mesenchymal markers N-cadherin and MMP11, resulting in induction of the EMT process.

Altogether, these results suggest that NF-κB acts directly to promote an aggressive phenotype of breast cancer cells through the transcriptional activation of EMT regulator genes. Our findings may contribute to a greater understanding and identification of the main players involved in the metastatic process in this tumor type. A simple model of the main findings of this work is shown schematically in Fig 9. Therefore, our work helps to reinforce NF-κB as a promising target for cancer therapy, especially for the management of invasive breast cancer.

## Supporting Information

S1 Table Primer sequences of the investigated genes. (DOC)

S2 Table Primer sequences flanking the predicted NF-κB binding sites. (DOC)

S1 Fig **NF-κB-luciferase reporter activity comparing the dose-response effects of DHMEQ in MDA-MB-231 (A) HCC-1954 (B) and MCF-7 (C) cells after 16 h of treatment.** The firefly luciferase was normalized to the renilla vector, and the values are relative to the pGL3 (Mock) signal. The bar graphs represent the relative luciferase activities of each DHMEQ concentration (3, 10 and 30 μg/ml) in breast cancer cells. Each bar represents the mean ± SD. * = *p*<0.05, ** = *p*<0.01, *** = *p*<0.001.
(TIF)

S2 Fig DHMEQ effect on cell viability. The cell viability rate evaluating three treatment conditions (3, 10 and 30 μg/ml of DHMEQ for 24 and 48 h) in MDA-MB-231 (A), HCC-1954 (B) and MCF-7 cells (C) compared to non-treated cells (empty bars). Each bar represents the mean ± SD. * = *p*<0.05, ** = *p*<0.01.
(TIF)

S3 Fig Relative expression of the EMT-related genes and ChIP-qPCR of predicted NF-κB/p65 binding sites using MCF-7 cells. mRNA levels of *SNAIL1*, *SLUG*, *TWIST1*, and *SIP1* (A) together with EMT-phenotype markers, such as *E-CADHERIN*, *N-CADHERIN* and *MMP11* (B), were assessed after 8, 16 and 24 h of DHMEQ treatment. (C) The evaluation of EMT-related genes after genetic silencing of NF-κB/p65. (D) ChIP results of predicted NF-κB/p65 binding sites in *SNAIL1*, *SLUG*, *TWIST1* and *SIP1* promoter regions. The histograms set a fold-change of each site by comparing the IgG negative control to NF-κB/p65 antibodies with the natural and treated (DHMEQ) condition. The data were expressed as the mean ± SD.
(TIF)
