# Supplementary material for: NF-kappaB Is Involved in the Regulation of EMT Genes in Breast Cancer Cells
Source: PLoS One. 2017 Jan 20;12(1):e0169622. doi: 10.1371/journal.pone.0169622 (PMC5249109; doi:10.1371/journal.pone.0169622)
Supplement: S1 Table — (DOC) [file pone.0169622.s001.doc]

**SUPPORTING INFORMATION**

**S1 Table.** Primer sequences of investigated genes.

| **Primer** | **Sequence** |
| --- | --- |
| *ACTB* forward | 5’-TAC AAT GAG CTG CGT GTG G-3’ |
| *ACTB* reverse | 5’-TAG CAC AGC CTG GAT AGC AA-3’ |
| *GAPDH* forward | 5’-ATT CCA CCC ATGG CAA ATT C-3’ |
| *GAPDH* reverse | 5’-GGC GTG GAT GGG TCT TTC A-3’ |
| *SNAIL1* forward | 5’-TCG GAA GCC TAA CTA CAG CGA-3’ |
| *SNAIL1* reverse | 5’-AGA TGA GCA TTG GCA GCG AG-3’ |
| *SLUG* forward | 5’-AAG CAT TTC AAC GCC TCC AAA-3’ |
| *SLUG* reverse | 5’-GGA TCT CTG GTT GTG GTA TGA CA-3’ |
| *TWIST1* forward | 5’-GGC ACC ATC CTC ACA CCT CT -3’ |
| *TWIST1* reverse | 5’-TGG CTG ATT GGC ACG ACC T-3’ |
| *SIP1* forward | 5’- CCC TTC TGC GAC ATA AAT ACG A-3’ |
| *SIP1* reverse | 5’-TGT GAT TCA TGT GCT GCG AGT-3’ |
| *E-CAD* forward | 5’-TGG CGT CTG TAG GAA GGC A-3’ |
| *E-CAD* reverse | 5’-GGC TCT TTG ACC ACC GCT CT-3’ |
| *N-CAD* forward | 5’-ACC AGG ACT ATG ACT TGA GCC-3’ |
| *N-CAD* reverse | 5’-GGC GTG GAT GGG TCT TTC A-3’ |
| *VIM* forward | 5’-GCC AGA TGC GTG AAA TGG AA-3’ |
| *VIM* reverse | 5’- CTG TCC ATC TCT AGT TTC AAC CG-3’ |
| *MMP11* forward | 5'-CCT GGA GGC TGC AAC ATA CC-3’ |
| *MMP11* reverse | 5’- TAC AAT GGC TTT GGA GGA TAG CA-3' |
| NF-κB/p65 forward | 5’-GAC CTG AAT GCT GTG CGG C-3’ |
| NF-κB/p65 forward | 5’-ATC TTG AGC TCG GCA GTG TT-3’ |
|  |  |
