# Supplementary material for: NF-kappaB Is Involved in the Regulation of EMT Genes in Breast Cancer Cells
Source: PLoS One. 2017 Jan 20;12(1):e0169622. doi: 10.1371/journal.pone.0169622 (PMC5249109; doi:10.1371/journal.pone.0169622)
Supplement: S2 Table — (DOC) [file pone.0169622.s002.doc]

**SUPPORTING INFORMATION**

**S2 Table.** Primer sequences flanking predicted NF-κB binding sites.

| **Primer** | **Sequence** |
| --- | --- |
| *SNAIL*1 -223 bp forward | 5’-TGC GCC AGC GAA CCC-3’ |
| *SNAIL1* -82 bp reverse | 5’-TTT GTC ACC TCC GCG CCA-3’ |
| *SNAIL1* -484 bp forward | 5’-TTT CCC TCG TCA ATG CCA CGC-3’ |
| *SNAIL1* -383 bp reverse | 5’-ACA CCT GAC CTT CCG ACG C-3’ |
| *SLUG* -651 bp forward | 5’-GCA TTT CTT TCA AGC CAC CAT AGC-3’ |
| *SLUG* -443 bp reverse | 5’-GAG AGG TTT GCC TTG CAC AAA GAC-3’ |
| *SLUG* -795 bp forward | 5’-GAG AGA ATG TCC GGT GGT TCC A-3’ |
| *SLUG* -674 bp reverse | 5’-GCT ATG GTG GCT TGA AAG AAA TGC-3’ |
| *TWIST1* -128 bp forward | 5’-GGT TTG GGA GGA CGA ATTG TTA GAC C-3’ |
| *TWIST1* +29 bp reverse | 5’-GTC TGG GAG TTG GGC GAG A-3’ |
| *TWIST1* -396 bp forward | 5’-GCG AGA TGA GAC ATC ACC CAC T-3’ |
| *TWIST1* -153 bp reverse | 5’-GGG GTC TAA CAA TTC GTC CTC CC-3’ |
| *SIP1* -830 bp forward | 5’-CGA CAG AAG CGT CAC GTT GGA A-3’ |
| *SIP1* -664 bp reverse | 5’-GGA GAG CAT GAT ATT AAA CGG CAT GG-3’ |
| *SIP1* -1268 bp forward | 5’-GGT CCT CTT AAC ACC TCC ACC TTT-3’ |
| *SIP1* -1050 bp reverse | 5’-GCT GGT TTA CAC TGC ACA TAA TTG GA-3’ |
