# Supplementary figures and images for: NF-kappaB Is Involved in the Regulation of EMT Genes in Breast Cancer Cells
Source: PLoS One. 2017 Jan 20;12(1):e0169622. doi: 10.1371/journal.pone.0169622 (PMC5249109; doi:10.1371/journal.pone.0169622)

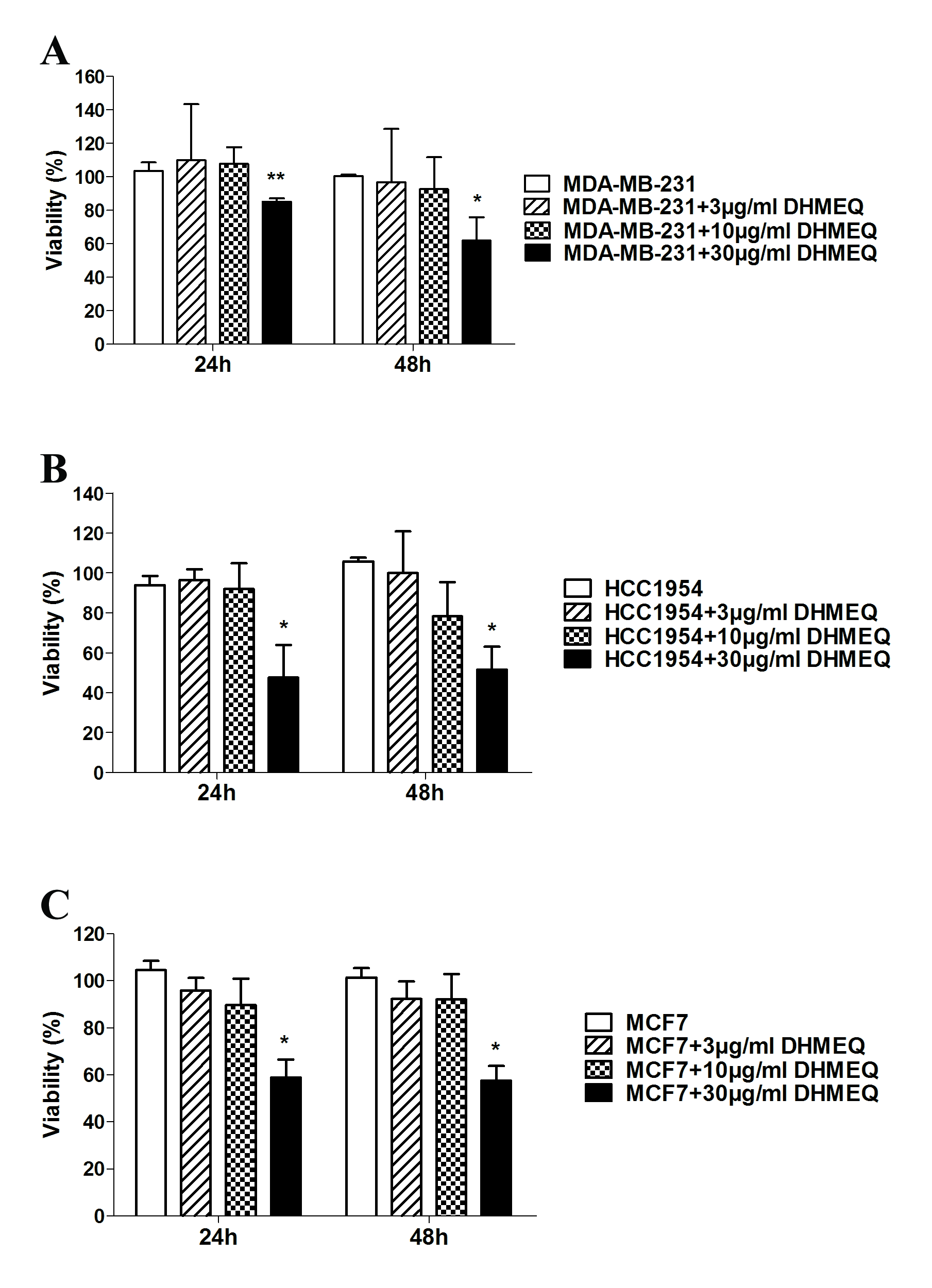

Supplement: S1 Fig — NF-κB-luciferase reporter activity comparing the dose-response effects of DHMEQ in MDA-MB-231 (A) HCC-1954 (B) and MCF-7 (C) cells after 16 h of treatment. The firefly luciferase was normalized to the renilla vector, and the values are relative to the pGL3 (Mock) signal. The bar graphs represent the relative luciferase activities of each DHMEQ concentration (3, 10 and 30 μg/ml) in breast cancer cells. Each bar represents the mean ± SD. * = p<0.05, ** = p<0.01, *** = p<0.001. (TIF) [file pone.0169622.s003.tif]

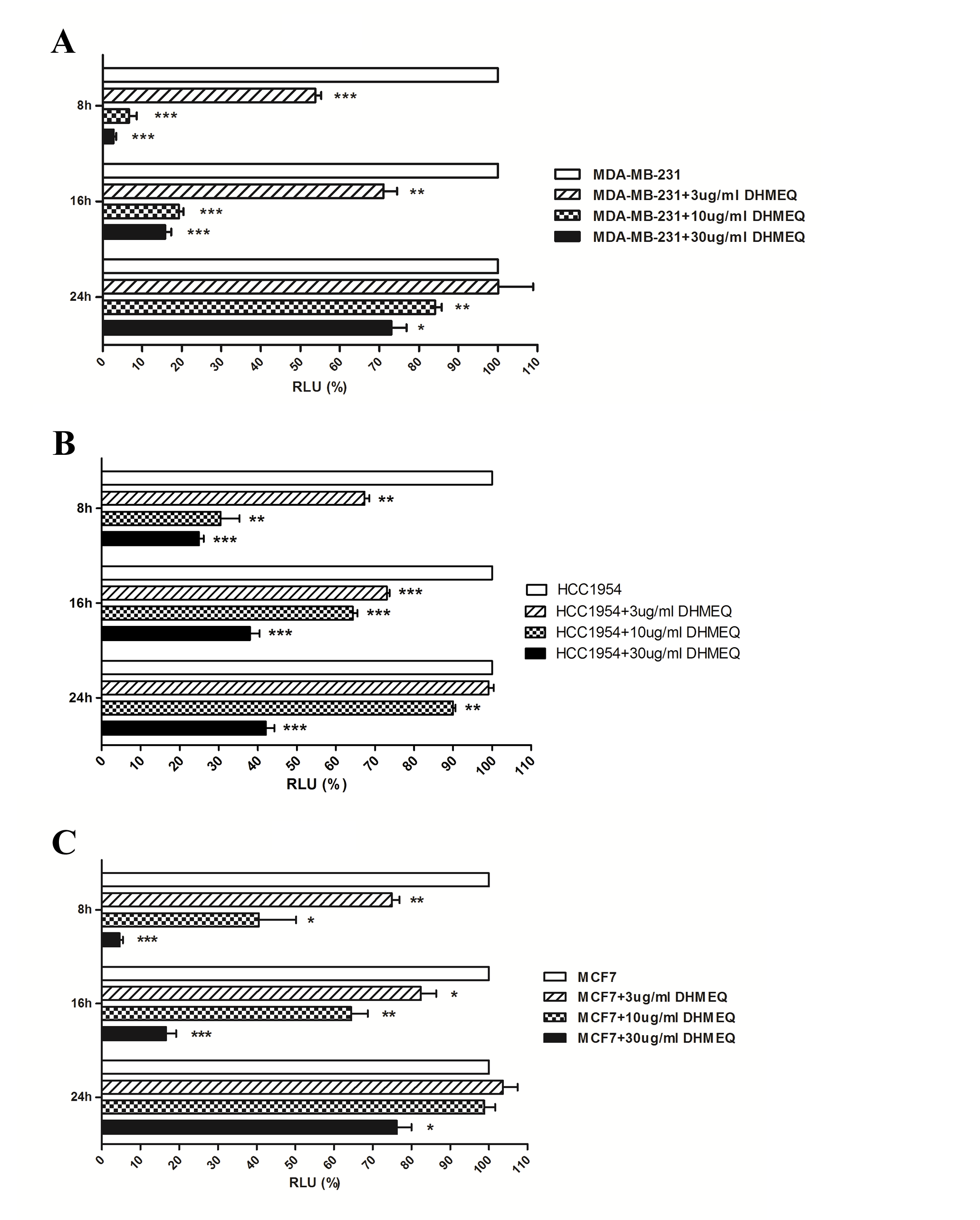

Supplement: S2 Fig — The cell viability rate evaluating three treatment conditions (3, 10 and 30 μg/ml of DHMEQ for 24 and 48 h) in MDA-MB-231 (A), HCC-1954 (B) and MCF-7 cells (C) compared to non-treated cells (empty bars). Each bar represents the mean ± SD. * = p<0.05, ** = p<0.01. (TIF) [file pone.0169622.s004.tif]

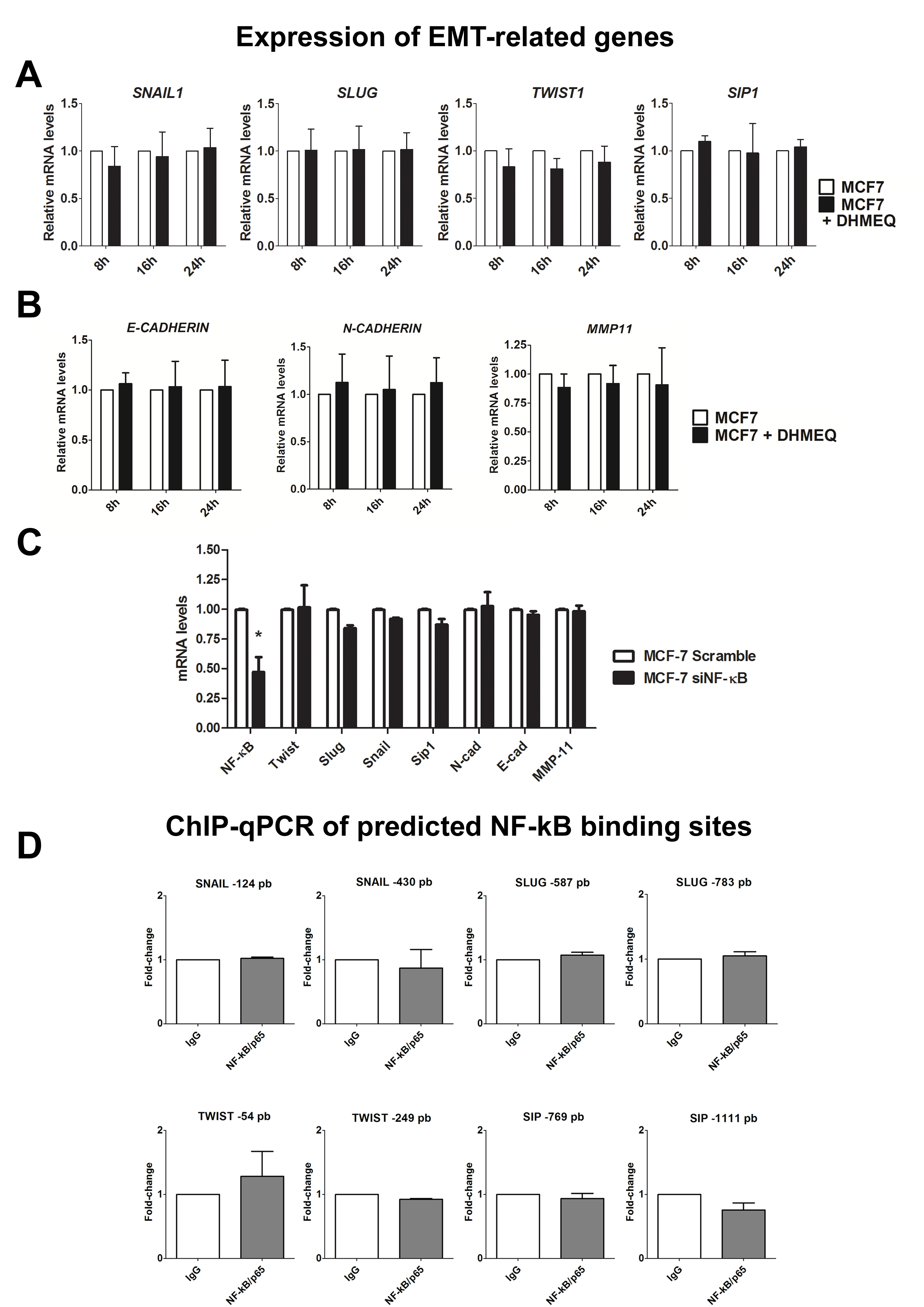

Supplement: S3 Fig — mRNA levels of SNAIL1, SLUG, TWIST1, and SIP1 (A) together with EMT-phenotype markers, such as E-CADHERIN, N-CADHERIN and MMP11 (B), were assessed after 8, 16 and 24 h of DHMEQ treatment. (C) The evaluation of EMT-related genes after genetic silencing of NF-κB/p65. (D) ChIP results of predicted NF-κB/p65 binding sites in SNAIL1, SLUG, TWIST1 and SIP1 promoter regions. The histograms set a fold-change of each site by comparing the IgG negative control to NF-κB/p65 antibodies with the natural and treated (DHMEQ) condition. The data were expressed as the mean ± SD. (TIF) [file pone.0169622.s005.tif]
